# How peoples’ ratings of dental implant treatment change over time?

**DOI:** 10.1007/s11136-019-02408-1

**Published:** 2020-01-06

**Authors:** Carolina Machuca, Mario V. Vettore, Peter G. Robinson

**Affiliations:** 1 School of Clinical Dentistry, University of Sheffield, Claremont Crescent, Sheffield, S10 2TA UK; 2 Bristol Dental School, University of Bristol, Lower Maudlin Street, Bristol, BS1 2LY UK

**Keywords:** OHRQoL, Response shift, Dental implants, Oral health

## Abstract

**Objectives:**

Dental implant treatment (DIT) improves peoples’ oral health-related quality of life (OHQoL). Assessment of longitudinal changes in OHRQoL may be undermined by response shift (RS). RS is the process by which quality of life changes, independent of health status as a result recalibration, reprioritization or reconceptualization. Thus, this study aimed to describe RS in the OHRQoL and perceived oral health of individuals receiving DIT and to compare the then-test, a self-anchored scale and the classification and regression trees (CRT) approaches for assessing RS.

**Methods:**

OHRQoL was assessed in 100 patients receiving DIT using the OHIP-Edent (*n* = 100) and a self-anchored scale (*n* = 45) before placement of the final restoration and 3 to 6 months after treatment was completed. The OHIP-Edent was also used as a retrospective assessment at follow-up. CRT examined changes in the OHIP-Edent total score as a dependent variable with global changes in oral health and each OHIP-Edent subscale score as independent variables.

**Results:**

OHRQoL and perceived oral health improved after treatment. The OHIP-Edent score decreased from 36.4 at baseline to 12.7 after treatment. On average, participants recalibrated their internal standard downwards (− 4.0 OHIP-Edent points). CRT detected downwards recalibration in 5% of participants and upwards in 15%. Reprioritization was observed in the social disability and psychological discomfort aspects of OHRQoL.

**Conclusions:**

RS affects longitudinal assessments of OHRQoL in DIT, reducing the apparent magnitude of change. The then-test and CRT are valid and complementary methods to assess RS.

**Electronic supplementary material:**

The online version of this article (10.1007/s11136-019-02408-1) contains supplementary material, which is available to authorized users.

## Introduction

Dental implants are a valuable treatment to replace teeth and are considered as an optimal solution to restore peoples’ mouths. The clinical success rate of dental implant treatment (DIT) is high; ranging from 73% for maxillary overdentures to 100% for mandibular single-tooth restorations, based on a minimum of 5 years of follow-up [1]. Likewise, peoples’ perception of this treatment is very good. Several studies report high levels of satisfaction and improved quality of life (QoL) with dental implant treatment [2]. However, even when all the clinical parameters are normal, patients report less improvement in areas such as pain or psychological aspects of oral health [3]. Furthermore, the initial improvements in Oral Health-Related Quality of Life (OHRQoL) may diminish when the treatment is reassessed over time [4, 5], perhaps because the benefits of treatment come to be taken for granted, or processes of coping and adaptation influence subjective assessments of health.

People can change their self-evaluations of QoL as a result of changes in internal standards (recalibration), values (reprioritization) and conceptualization (reconceptualization) also known as response shift (RS) [6]. The RS phenomenon challenges traditional assessments of change on QoL that use pre–post-test designs, and has been extensively studied in numerous health conditions [7–11].

Evidence shows that important benefits of dental treatments may be undetected if RS is not accounted for [12–16]. Kimura et al. [14] assessed RS as influencing apparent treatment efficacy in patients with DIT. Treatment efficacy of DIT was four times higher when RS was considered. Alternatively, RS may reduce the magnitude of improvement derived from treatment. Thus, in studies reporting change in OHRQoL as small or moderate, RS might have masked, under or overestimating the treatment benefit.

Therefore, it is important to take RS into account when OHRQoL is assessed as an outcome of treatment efficacy. The most common method to assess RS is the then-test, which constitutes a retrospective judgment of pre-test internal standards at the time of the post-test [6, 17]. Additionally, the ideal scale approach incorporates an ideal criterion to assess QoL and has been used to assess RS in dental patients with interesting results [15]. Statistical methods to assess RS include classification and regression trees (CRT), which has successfully analysed complex interactions between variables [18, 19].

Thus, this study aimed to describe RS in the OHRQoL and perceived oral health of individuals receiving DIT. Because one single method may not be sufficient to assess the different components of RS, we compared three approaches: the then-test, the self-scale anchored approach and the classification and regression trees. Furthermore, the convergent validity of the three methods was explored to determine their relative utility.

## Methods

### Participants

This longitudinal study assessed changes in OHRQoL in edentulous (partial or total) adults. All patients aged 16 years and above, requiring restorative treatment after dental implant placement, referred to the Academic Unit of Restorative Dentistry of the Charles Clifford Dental Hospital (CCDH), Sheffield UK, were included. CCDH dental services are partially covered by the NHS (National Health Service), which is the publicly funded national healthcare system in the UK. Exclusion criteria were patients below 16 years old or not eligible for DIT.

The recommended sample size when using classification and regression trees (CRT) is ten events per variable to obtain a reasonably predictive model with stable performance [20, 21]. The analysis used the seven subscales of the OHIP-Edent as the independent variables and four predictors of the magnitude and direction of RS (gender, number, position of replaced teeth and treatment modality). With those 11 independent variables, the intended sample size was 110 participants. Due to the nature of treatment, modest loss to follow-up was anticipated and 20% more participants were recruited. Therefore, the inception cohort was 132 participants.

Participants provided written consent and completed the baseline questionnaires on their first appointment for the definitive restorative treatment. A second set of questionnaires was given at their routine post-restorative treatment review 3 to 6 months after completion.

The study was approved by the National Ethics Research Committee Service (NRES) Yorkshire and The Humber (STH ref STH18703; REC ref 14/YH/1320).

### Patient-reported outcomes

OHRQoL was assessed using a shortened version of the Oral Health Impact Profile for edentulous patients (OHIP-EDENT). Perceived oral health was measured using a self-anchored scale and a global rating of oral health.

The OHIP-Edent measures aspects of OHRQoL influenced by edentulousness and its treatment [22]. The questionnaire has 19 questions on seven subscales: functional limitation, physical pain, psychological discomfort, physical disability, psychological disability, social disability and handicap. Participants rate their oral health problems for each item on a 5-point Likert scale [Never (0) to Very often (4)]. A summary measure is calculated as the sum of the scores for each participant (possible range 0–76). Higher scores indicate worse OHRQoL. OHIP-Edent detects clinically relevant change in individuals with DIT [22] with good internal consistency and validity [23, 24]. The questionnaire was administered to the complete sample at baseline and follow-up. At follow-up, participants were also asked to judge (‘then’) their OHRQoL at the time of the first interview retrospectively.

**Fig. 1:**
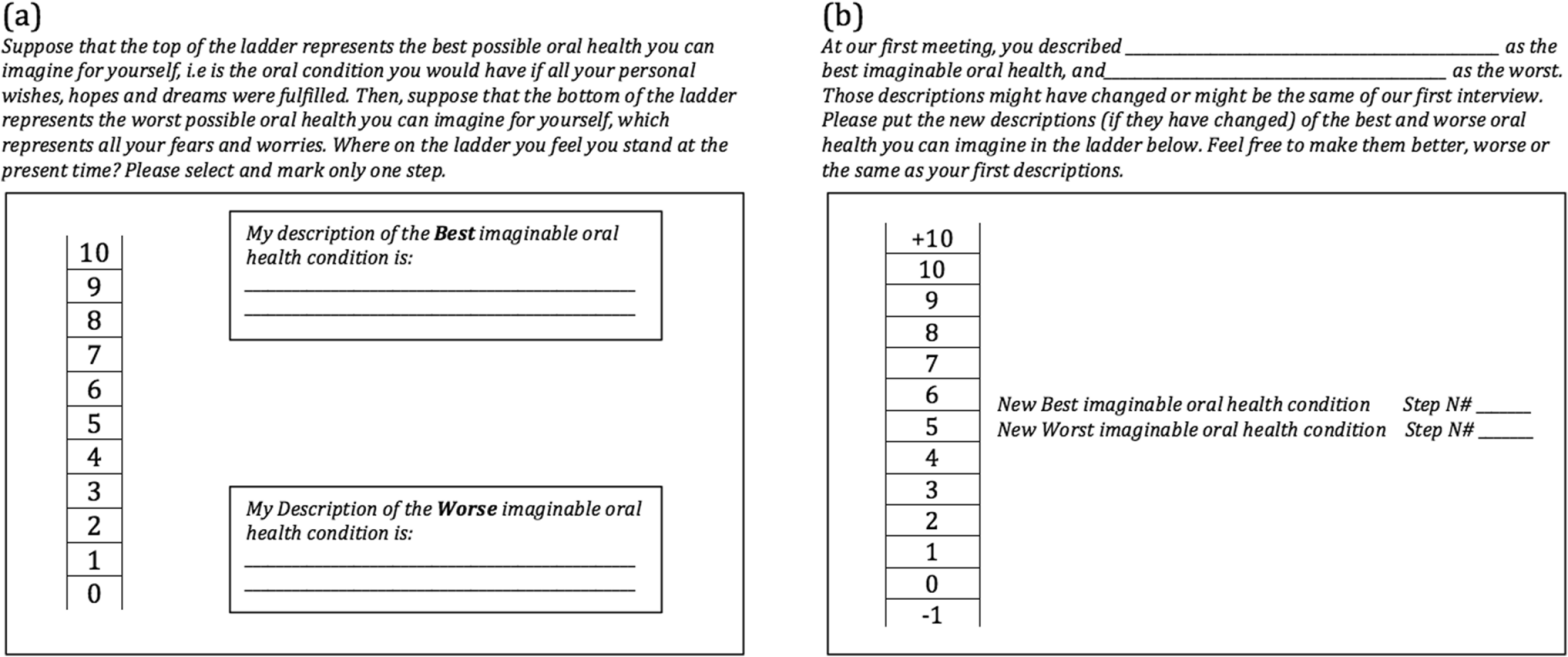
Self-anchored scale. **a** The rating is presented as a ladder with the bottom marked with the number 0 symbolizing the worst and 10 the best. Each participant was asked to situate her or himself in the present. **b** At follow-up, participants again described the best and the worst imaginable oral health and located themselves on the ladder. The new descriptors (if there were any) could be rated even worse, better or coinciding with the descriptions provided at baseline

The self-anchored scale was designed as an individualized variant of the ideals scale [25] to assess perceived oral health and RS and was based on the Cantril’s ladder [26]. At baseline, participants were asked to provide a written description of the ‘best’ and ‘worst’ possible oral health state for them in order to establish the scale anchors at the bottom and top of the ladder (Fig. 1a). Participants then specified where on the ladder they were. At follow-up, participants again described their best and worst imaginable oral health and located themselves on the ladder. The new descriptors (if there were any) could be rated worse, better or coinciding with the descriptions provided at baseline (Fig. 1b).

The global rating of oral health (“Overall, how would you rate the health of your mouth, teeth and gums?”) and transition judgement questions (“Overall, how has your oral health changed since our last meeting?”) were used to assess patients’ perceived oral health and the extent to which had it changed since baseline, respectively.

### Data analysis

Descriptive analysis summarized demographic variables (age, gender), treatment characteristics (number and position of replaced teeth, treatment modality), OHRQoL (OHIP-Edent) and perceived oral health (self-anchored scale and global rating of oral health) at baseline and follow-up.

The reliability of the OHIP-Edent was assessed using internal consistency by calculating the OHIP-Edent total score and subscales *Cronbach* α statistics. Test–retest reliability was assessed by calculating the OHIP-Edent total score at baseline and follow-up intra-class correlation coefficient (ICC). The Spearman rank correlation between the OHIP-Edent total score and the global ratings of oral health was used to assess its convergent validity.

Changes in OHRQoL and perceived oral health were analysed depending on the approach used to assess RS, as described below.

#### The then-test

Schwartz and Sprangers recommendations for the then-test use were applied in this study [17]. The OHRQoL observed change (unadjusted change) was calculated as OHIP-Edent follow-up minus the baseline score. Recalibration RS was calculated as the OHIP-Edent then-test score minus the baseline score. The true change (adjusted change) was calculated as the difference between the OHIP-Edent follow-up and the then-test score. A negative sign of recalibration suggests participants retrospectively reassessed their baseline OHRQoL as having less impact than they thought at baseline. Thus, such participants changed their internal standards downwards. A positive sign of recalibration may indicate that people retrospectively assessed their baseline status as worse than they thought at baseline, i.e. they changed their internal standards upwards.

The analysed data was not normally distributed. Therefore, hypotheses tests for true and observed change and recalibration were conducted using the nonparametric Wilcoxon signed rank test. The recalibration effect size (ES) was calculated as:

$${\text{ES}} = \frac{Z}{\sqrt N },$$

where *Z* is the *z* statistic and *N* is the number of observations.

Guidelines suggest that an ES 0.1 is small, 0.3 is medium, and > 0.5 is large [27].

The predictor variables influencing the magnitude and direction of RS were investigated after normalization of the data, using multiple linear regressions with recalibration as the outcome variable and gender, number and position of replaced teeth and treatment modality as independent variables. Further sociodemographic variables were not included due to sample limitations.

#### Self-anchored scale

Recalibration of perceived oral health was calculated as the difference between baseline and transformed baseline scores. True change was calculated as the difference between follow-up and transformed baseline scores. The transformed scores are function of the baseline scores and the position of the best and worse new anchors in the Cantril’s ladder at follow-up [25]:

$$X_{\text{trans}} = \, \left( {\left( {B_{{{\text{follow}}{-}{\text{ up}}}} {-} \, W_{{{\text{follow}}{-}{\text{ up}}}} } \right) \, X_{\text{baseline}} + \, 10W_{{{\text{follow}}{-}{\text{ up}}}} {-} \, B_{{{\text{follow}}{-}{\text{ up}}}} } \right)/9,$$

where *X*_baseline_ baseline self-anchor scale score, *B*_follow-up_ best imaginable oral health anchor at follow-up, *W*_follow-up_ worse imaginable oral health anchor at follow-up.

The ES for the self–anchored scale was calculated using recalibration as a function of the standard deviation:

$$\begin{aligned} {\text{Recalibration }}\;\;{\text{ES}} & = \frac{{{\text{True }}\;{\text{change }}{-}{\text{ Observed }}\;{\text{change}}}}{{{\text{SD}}\;{\text{ Observed }}\;{\text{change}}}} \\ & = \frac{{X_{{\text{follow}}{-}{\text{ up}}} {-} \, X_{\text{trans}} ) \, {-} \, \left( {X_{{\text{follow}}{-}{\text{ up}}} {-} \, X_{\text{baseline}} } \right)}}{{{\text{SD}}_{{\text{follow}}{-}{\text{ up}}} -_{\text{baseline}} }}. \\ \end{aligned}$$

As with the then-test, an ES 0.1 is small, 0.3 is medium, and > 0.5 is large [27].

Ceiling and floor effects occur when many participants score the maximum or minimum scores. The proportion of participants who achieved each possible score of the self-anchored scale (within range 0–10) was analysed at baseline and follow-up. Ceiling and the floor effects were defined as present if 15% or more participants achieved the maximum or minimum score [28].

#### Classification and regression trees (CRT)

CRT is a representation where each member of the studied population is classified based on several independent variables [29]. The terminal nodes or tree leaves represent a cell of a partition and have a simple model attached which applies to that cell only. The CRTs were created using the OHIP-Edent observed change score (OHIP-Edent follow-up − OHIP-Edent baseline) as the dependent variable with the observed changes of the seven subscales as covariates to explore change patterns in the subscale scores.

Using these independent variables in a regression model, each node is split through the conceptually important and the most strongly predictive independent variable, maximizing the purity of the resulting nodes; a node is considered ‘pure’ when all the cases have the same value for the dependant variable. If the primary splitting variable is missing for an individual observation, this information is not discarded but instead, a surrogate variable that has the best similar pattern relative to the outcome variable is used, thereby enabling utilization of incomplete datasets.

As a result of the ‘surrogates’ in splitting the data, the contribution that a variable can make to the model is not only determined by the primary splits. A variable can be considered as highly important even when it does not appear as a node splitter. The CRT method explores the improvement measure attributable to each variable in its role as either a primary or surrogate splitter. The values of all these improvements are summed over each node and totalled to determine the ‘variable importance’. Then, they are scaled relative to the best performing variable; the variable with the highest sum of improvement is scored 100 and all the other will have proportionally lower scores [30].

The following criteria were used in the analysis [31]:

- Parent node minimum number of cases: 10% of the sample.
- Stopping rule for a terminal node: 5% of the sample.
- Tenfold cross-validation to validate the tree.
- Tree pruning to avoid over fitting with a maximum acceptable difference in risk between the pruned and the sub-tree of 1 standard error.
- Missing data handled by surrogate splits.

The model performance was investigated calculating the risk estimate. The risk estimated is a measure of within-node variance and can be used as a criterion of model fit. Lower values indicate a better model. Thus, the model fit was calculated following this formula:

$$S^{2}_{\text{e}} = \frac{{{\text{Risk }}\;{\text{value}}}}{{S^{2}_{y} }} ,$$

where $S^{2}_{\text{e}}$ error variance or proportion of variance due to error, *Risk value* variance within node, $S^{2}_{\text{y}}$ dependent variable or root node variance or standard deviation of the root node squared

Thus, the variation in the dependent variable explained by the model ($S^{2}_{\text{x}}$) or explained variance is $S^{2}_{\text{x}} = 1{-}S^{2}_{\text{e}}$.

#### Operationalization of response shift in the CRT model

Response shift was operationalized as described in Table 1. Recalibration of OHRQoL was inferred when the global transition judgement was inconsistent with the observed OHIP-Edent change score. If participants reported better OHRQoL at follow-up but their global transition judgement remained unimproved, this was interpreted as upward recalibration. If participants reported worse OHRQoL at follow-up but rated their global oral health as improved, then downward recalibration was inferred.

**Table 1:** Operationalization of response shift in the CRT model

Response shift	Operationalization	Qualitative indicator	Interpretation
Recalibration	Changes in subscale scores over time	↓ OHIP-Edent scores at follow-up with global rating oral health unimproved	Upward recalibration At follow-up, individuals state global oral health as unimproved but rated their QoL as better at follow-up [follow-up − baseline = (−)]
		↑ OHIP-Edent scores at follow-up with global rating oral health improved	Downward recalibration At follow-up, individuals state global oral health as improved but rated their QoL as worse at follow-up [follow-up − baseline = (+)]
		↓ OHIP-Edent scores at follow-up with global rating oral health improved	No recalibration At follow-up, individuals state global oral health as improved and rated their health as better at follow-up [follow-up − baseline = (−)]
Reprioritization	Changes in the relative importance of each subscale to the model over time		

Reprioritization was inferred from changes in the variable importance of the domains of OHIP-Edent before and after the treatment.

This study reports the full rather than the pruned tree because, especially in small samples, pruning is likely to omit small groups or participants with subtle changes in QoL [19].

The OHIP-Edent minimal important difference (MID) was considered as a threshold for interpretation of change. MID is defined as the smallest difference in score in the domain of interest which patients perceive as beneficial [32]. Thus, any variation above or below the MID threshold would influence the assessment of quality of life over time and interpreted as recalibration. MID for the OHIP-Edent has been considered as 9 points [33]. This value was used to identify clusters of participants potentially with RS.

## Results

A total of 140 partially or total edentulous adults aged 16 years and above were invited to participate. Of these, 127 enrolled for the study between March 2015 and June 2016. Participants’ failure to attend for the last review was the only reason for loss to follow-up (*N* = 27). Details are listed in a supplementary online file. The amount of missing information was 12% of all OHIP-Edent items. Thus, not all the data are complete, and each analysis is reported with the number of data available.

The mean age of the 127 participants who completed the OHIP-Edent at baseline was 37.5 (SD + 16.9) years and 57.5% were female. From them, 98 completed the follow-up (78.7%). The self-anchored scale was completed by 121 participants at baseline (Mean age 37.3 ± 16.8; 57.9% female), but only 45 (37.2%) at follow-up (Mean age 38.9 ± 17.2; 62.7% female). Thus, the subsequent data analysis of this construct was for descriptive purposes only.

Participants were treated with implant-supported crowns (ISC), implant-supported bridges (ISB) or implant-supported overdentures (OD). Seventy-eight participants (62.2%) were treated with single upper anterior implant-supported crowns (Table 2).

**Table 2:** Participants’ clinical characteristics at baseline

Variable	*n*	All participants (*n* = 127) %
Treatment characteristics		
Number of patients by number of replaced teeth		
1	73	57.5
2	25	19.7
≥ 3	29	22.8
Arch of replaced teeth (U/L)		
Upper	106	83.5
Lower	19	15.0
Both	2	1.6
Position of replaced teeth (A/P)		
Anterior	84	66.1
Posterior	21	16.5
Both	22	17.3
Treatment modality		
Implant-supported crown	99	78.0
Implant-supported bridge	13	10.2
Implant-supported overdenture	15	11.8

Table 3 presents OHIP-Edent scores and self-anchored scale before and after the restorative treatment. The mean OHIP-Edent score at baseline was 36.4 and decreased to 12.6 at follow-up. The mean self-anchored scale scores were 6.2 and 7.7 before and after treatment, respectively. At baseline, 33.3% of participants rated their oral health as ‘excellent’ or ‘very good’, the corresponding figure at follow-up was 66.4%.

**Table 3:** Participants’ oral health-related quality of life (OHIP-Edent total and subscale scores) and perceived oral health (global ratings of oral health and self-anchored scale) at baseline, follow-up and then-test/transformed scores

	Baseline	Follow-up	Then-test or transformed baseline
OHIP-Edent *N* = (98–127)			
Functional limitations	6.7 (3.5)	2.3 (2.4)	5.7 (3.9)
Physical pain	6.8 (4.6)	2.5 (3.0)	6.2 (4.9)
Psychological discomfort	5.4 (2.2)	2.5 (2.4)	4.8 (2.6)
Physical disability	5.6 (3.9)	1.7 (2.6)	5.1 (4.1)
Psychological disability	4.8 (2.7)	1.6 (2.0)	4.3 (2.8)
Social disability	3.8 (3.8)	1.1 (2.3)	3.4 (3.8)
Handicap	3.3 (2.7)	0.8 (1.7)	2.8 (2.9)
Total	36.4 (20.5)	12.6 (14.0)	32.4 (21.9)
Self-anchored scale *N* = (45–121)			
Total	6.2 (2.3)	7.7 (1.5)	6.4 (2.4)
Global ratings of oral health *N* = (98–126)			
Excellent	9 (7.1)	25 (24.8)	12 (12.2)
Very good	33 (26.2)	42 (41.6)	21 (21.4)
Good	72 (57.1)	30 (29.7)	36 (36.7)
Poor	10 (7.9)	4 (4.0)	22 (22.4)
Very poor	2 (1.6)	0	7 (7.1)

Values are shown as OHIP-Edent and self-anchored scale mean (SD) and global ratings of oral health

OHIP-Edent internal consistency, assessed using Cronbach’s α and by correlations between OHIP-Edent total scores and each subscale all exceeded 0.7 and were significant at *p* < 0.05, indicates acceptable internal consistency. When individual items were deleted, the alphas remained stable. The test–retest reliability, assessed by intra-class correlation coefficients (ICC) for OHIP-Edent baseline and follow-up, was 0.543 which indicates moderate reliability [34]. Convergent validity between the OHIP-Edent total score and the global rating of oral health was small but non-significant for the baseline assessment (*r*_Spearman_ = 0.13; *p* = 0.18) and medium for the follow-up (*r*_Spearman_ = 0.30; *p* < 0.01).

### Response shift

#### Then-test

**Fig. 2:**
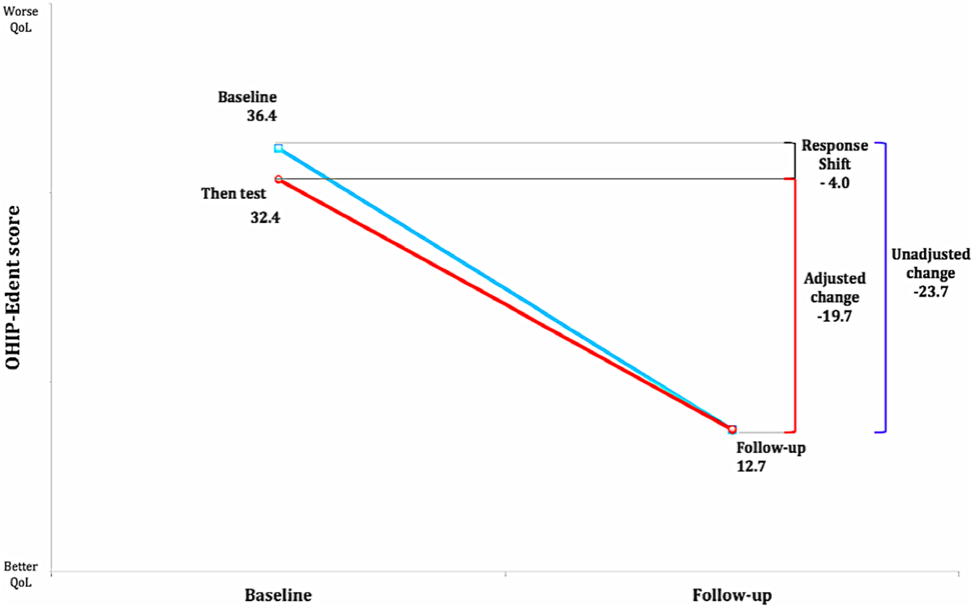
Recalibration in the then-test approach

The then-test was completed by 98 participants who retrospectively assessed their baseline OHRQoL as better (i.e. lower OHIP-Edent scores) than they had at baseline (Fig. 2). The negative sign of this recalibration suggests that, on average, participants recalibrated downwards. The overall magnitude of RS was − 4.0 ± 15.3 OHIP-Edent points with a small ES (*p *< 0.05; Wilcoxon Sign Rank test) (Table 4).

**Table 4:** OHIP-Edent mean scores at baseline, follow-up and then-test, recalibration response shift, observed change, true change by treatment modality

Treatment modality	*n*	Baseline	Follow-up	Then-test	Recalibration response shift	Effect size	*p* value	Observed change	Effect size	*p* value	True change	Effect size	*p* value
ISC	72	31.5 (19.2)	10.3 (10.5)	28.8 (20.1)	− 2.7 (14.6)	0.2	0.15	− 21.2 (19.3)	0.8	0.001*	− 18.5 (19.8)	0.8	0.01*
ISB	12	41.6 (18.7)	19.7 (17.8)	38.8 (24.2)	− 2.9 (10.1)	0.3	0.32	− 22.0 (10.0)	0.8	0.002*	− 19.1 (17.4)	0.8	0.01*
OD	14	56.9 (14.0)	18.7 (22.2)	45.3 (24.0)	− 11.6 (21.2)	0.5	0.05*	− 38.2 (22.1)	0.9	0.001*	− 26.6 (26.0)	0.9	0.01*
Total	98	36.4 (20.5)	12.7 (14.1)	32.4 (22.8)	− 4.0 (15.3)	0.2	0.02*	− 23.7 (19.6)	0.8	0.001*	− 19.7 (20.6)	0.8	0.01*

Values are shown as mean (SD)

*ISC* implant-supported crowns, *ISB* implant-supported bridges, *OD* implant-supported overdentures

*Statistically significant at *p* < 0.05 Wilcoxon signed rank test (two tailed)

Adjusting for recalibration (true change) reduced the magnitude of change for all types of treatment with a large ES. The value of true change was significantly reduced in the OD group with a difference of 12 OHIP-Edent points (Table 4).

Gender, the number and position of replaced teeth and treatment modality did not predict recalibration (*R*^2^ = 0.66; *F*(5,89) = 1.25, *p *= 0.29) (Table 5).

**Table 5:** Multiple linear regression analysis of predictors of recalibration

Predictor variables	SRC	SE	*p* value
Gender (female/male)	0.049	3.38	0.641
Number replaced teeth	− 0.362	1.03	0.298
Position replaced teeth (upper/lower/both)	0.161	4.86	0.264
Position replaced teeth (anterior/posterior/both)	− 0.188	3.28	0.286
Treatment modality (ISC/ISB/OD)	0.217	6.79	0.501

*N* = 98; *R*^2^ = 0.066

*SRC* standard regression coefficient

#### Self-anchored scale

Overall, the self-anchored scale (*n* = 45) revealed significant improvement in participants’ perceived oral health (observed change mean = 1.7; SD = 2.3) with a large ES (ES = 0.74; *p* < 0.001). Using the self-anchored scale, recalibration was very small and non-significant (recalibration mean = 0.1; SD = 1.2). Thus, the true change (true change mean = 1.4; SD = 2.0) was very similar to the observed change, with a strong and significant effect (ES = 0.61; *p* = < 0.001).

There were no floor or ceiling effects for the self-anchored scale scores at baseline or follow-up. At baseline, only 1.6% of participants achieved the worst score (0) and 8.2% the best (10). After treatment, no participants achieved the worst score (0) and 10.3% achieved the best (10). However, there were floor and ceiling effects for the anchors. Most participants rated the best and the worse descriptors of oral health as the endpoints of the scale. At baseline, 57.9% of participants rated the worst descriptor with the worst score (0) and 76.8% rated the best descriptor with the best score (10). After treatment, 62.3% of participants rated the worst descriptor with the worst score (0) and 77.9% rated the best descriptor with the best score (10).

#### Classification and regression trees

**Fig. 3:**
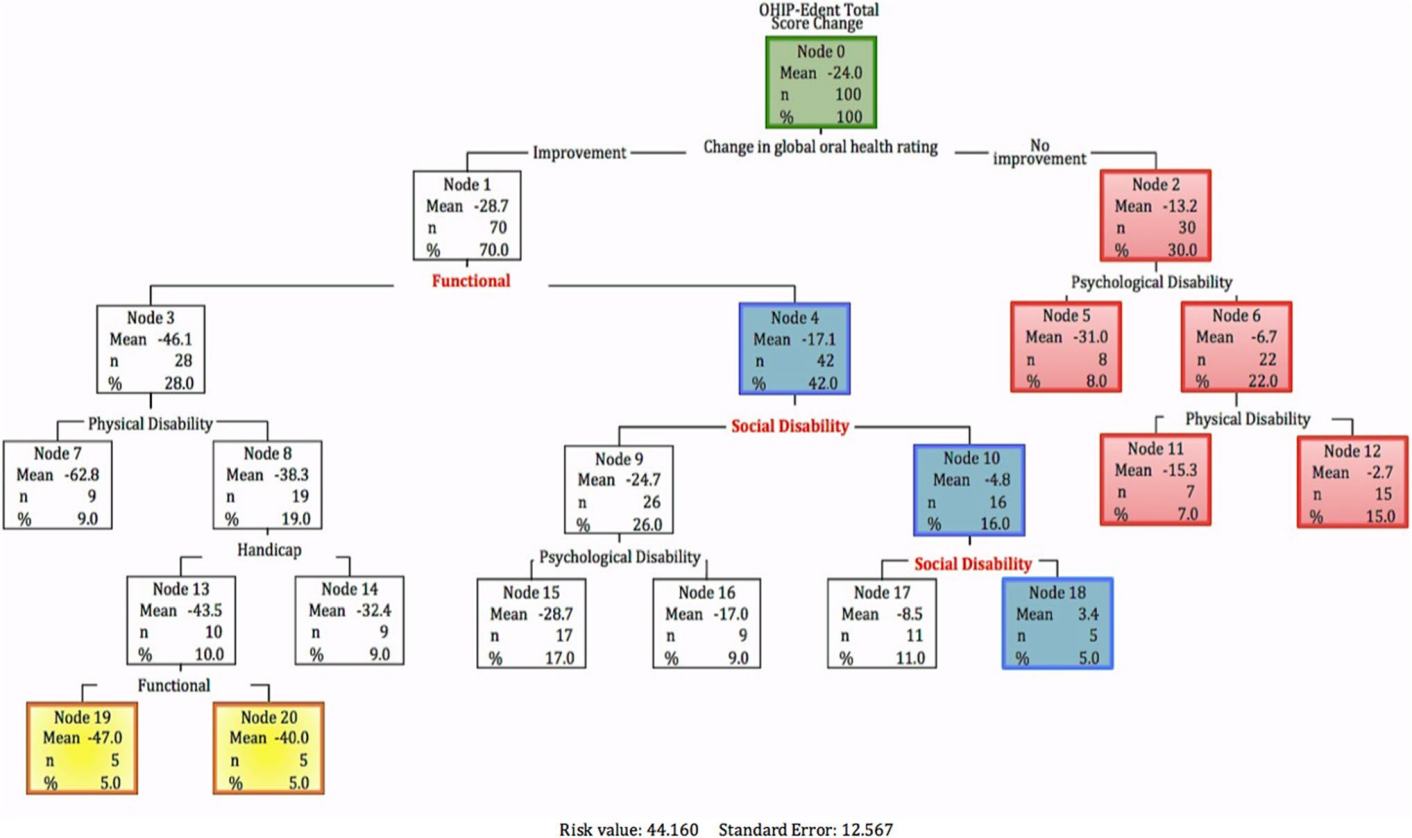
Classification Tree among 100 participants receiving implant-supported prostheses

CRT analysis was conducted with participants who completed the OHIP-Edent before and after the treatment (*n* = 100). The sample was classified first using their global transition judgement (Fig. 3). Thus, participants at the follow-up rating their global oral health as ‘much better’ and ‘better’ were categorized as reporting ‘improvement’ (Node 1) and those rating their global oral health as ‘about the same’, ‘worse’ and ‘much worse’ as ‘no improvement’ (Node 2). The tree was fitted using OHIP-Edent total change score as the dependent variable and the change of the seven subscales as independent variables.

Model performance was assessed calculating the risk estimate. The proportion of variance due to error was 0.12. The variation in the dependent variable explained by the model ($S^{2}_{\text{x}}$) was 0.88. Thus, 88% of the variation in OHIP-Edent total score was explained by the variation in subscales scores, which had a strong effect in forming the tree.

#### Tree analysis

Overall, 70% (Node 1) of participants reported an improvement in their oral health after treatment.

Among those whose global transition judgement indicated ‘improvement’, the second split was defined by changes in functional limitations (Nodes 3 and 4). Whereas for those whose global transition judgement did not improve, the second split was defined by changes in psychological disability (Nodes 5 and 6). Changes in physical disability (Node 7, 8, 11 and 12), social disability (Nodes 9, 10, 17 and 18), handicap (Nodes 13 and 14), functional disability (Nodes 19 and 20) and psychological disability (Nodes 15 and 16) defined the succeeding splits.

Recalibration was inferred when the global rating of oral health change was inconsistent with the OHIP-Edent change score. Overall, participants reporting an improvement in their oral health after treatment showed larger mean total scores for OHIP-Edent, but 5% of them (Node 18) rated their QoL as worse at follow-up. Node 18 indicates that this might be because social aspects of their oral health remained unimproved (downward recalibration).

The right side of the tree (Node 2) shows that nearly one-third of participants manifested no change in their global transition judgement even when they rated their QoL as better at follow-up, as indicated by the negative sign of the mean scores (upward recalibration). The 15 participants represented in the terminal Node 12 reached the MID of 9 points, which was used as the threshold to detect recalibration.

**Fig. 4:**
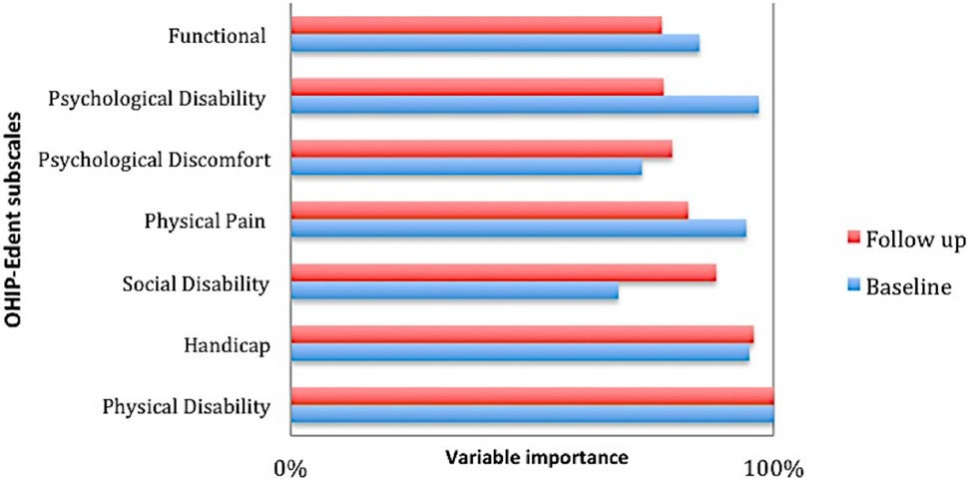
Variable importance at baseline and follow-up

Reprioritization was inferred from changes in the order of importance of the OHIP-Edent subscales before and after the treatment. In this model, the social disability and psychological discomfort aspects of QoL increased in importance over time (Fig. 4).

#### Comparing methods

Despite the low completion rates of the self-anchored scale, the results of the three methods are comparable. All three approaches detected RS. Using the then-test, participants on average recalibrated downwards and with the CRT, downward recalibration can be inferred in participants in node 18 (Fig. 3).

Adjusting for recalibration reduced the magnitude of change. Using the then-test, the magnitude of improvement on OHRQoL was reduced by 4.0 OHIP-Edent points and the magnitude of perceived oral health was reduced by 0.3 points using the self-anchored scale.

CRT demonstrates changes in the patterns of RS compatible with those obtained with the then-test and the self-anchored scale. The magnitude of improvement in OHRQoL is reduced among participants who rated their general oral health as not improved (Node 2) and is reduced when they recalibrated downwards (Node 18) (Fig. 3).

The subscales where recalibration occurred were comparable between the then-test and the CRT. The then-test detected downward recalibration with a significant change on the functional limitations subscale (Table 3). The CRT detected downward recalibration influenced by functional changes as observed in the first split of the tree differentiating those participants with improvement of their QoL (Node 1) with the highest (Node 3) and the lowest ratings of QoL (Node 4) (Fig. 3).

## Discussion

This study described RS in OHRQoL and perceived oral health in individuals after DIT supported prostheses and compared three approaches to detect it: the then-test, a self-anchored scale and the classification and regression trees.

The then-test detected recalibration. Overall, the negative sign of the recalibration score indicates that participants retrospectively reassessed themselves as having less impact on their OHRQoL than they thought at baseline. This result may be interpreted as participants reducing their internal standards or downward recalibration and implies that the magnitude of improvement on OHRQoL is reduced when adjusting for RS. This finding has not been reported in the oral health literature or has been encountered only infrequently [15]. RS has been defined as adaptation to changed health. From this perspective, if individuals have deteriorating conditions, they might decrease their internal standards to accommodate the illness and maintain acceptable QoL. This had been reported in several studies where individuals with declining health conditions as cancer or multiple sclerosis retrospectively assessed their QoL as better as a way of adapting to their status [35, 36]. Conversely, despite the dental treatment having a positive impact on OHRQoL and our participants’ oral health improving, they also retrospectively reassessed their baseline OHQoL as better. A potential explanation for this finding is that participants may have overestimated the impact of DIT on OHRQoL at baseline. Then, when they reassessed these impacts retrospectively made more positive assessments of their previous OHRQoL. Furthermore, this explanation leads to another cause, effort justification bias. Since participants had invested time and other resources (for example, the surgical aspects of their DIT), they might initially have overestimated their level of impacts to justify undergoing the treatment.

Participants’ expectations is yet another possible explanation. Several studies have reported that patients have unrealistic expectations of DIT [4, 37, 38]. Dental implants are considered by some individuals as the solution to all their dental problems, restoring appearance, function and QoL to absolute normality [39]. Treatment expectations among participants in this study might have been high; therefore, in retrospect and based on their post-treatment state, they reassessed their OHRQoL better than at baseline because treatment had not fulfilled these expectations.

The overall magnitude of recalibration RS was − 4.0 ± 15.3 OHIP-Edent points with a small ES. A small ES for recalibration when using the then-test has been reported in serious [25] and mild health conditions [15]. However, RS should be considered when assessing change because even a small recalibration may result in a misrepresentation of the true change in QoL [35]. Participants with objective improvement (for example, assessed clinically) may report no increase in OHRQoL due to recalibration. Considering recalibration in this way may make such an improvement appreciable.

The second approach used to assess recalibration was the self-anchored scale. Only 45 (37.2%) of the 121 participants completed both assessments. This represents a huge loss of data. The self-anchored scale contains open-ended questions; therefore, the effort required to complete the task is high. The cognitive burden of defining the anchors was difficult for some participants, which may also explain the high attrition rate.

While these drawbacks undermine the potential usefulness of this approach, the results obtained from the 45 participants with complete data are intuitive. They show a significant improvement in perceived oral health with a large ES. Nonetheless, as with other ideal measures, the self-anchored scale showed floor and ceiling effects when participants indicated the endpoints of the scale (0 and 10, respectively) at both baseline and follow-up. When participants select the endpoints of the scale as their anchors, there it may be little scope recalibration to be detected [40].

The third approach, the CRT, may be a useful method to investigate patterns of RS because of its graphical representation of the data (Fig. 3). Apparently, and observing the subscale trajectories across the tree, if functional changes remained unimproved (Node 1), then participants reported worse OHRQoL due to changes in social aspects of oral health (Nodes 4,10,18). It seems reasonable to consider that, if the functional (or aesthetic aspects) of the treatment have not been fulfilled, this inevitably will restrict improvements on the social aspects of OHRQoL.

The right side of the tree shows that 30% of participants (Node 2) indicated no change in perceived oral health but rated their OHRQoL as better after the treatment; of those, 15 reached the MID of 9 OHIP-Edent points (Node 12). According to the operationalization proposed, this corresponds to upward recalibration. This finding has two possible explanations. First, these individuals may have recalibrated their internal standards upwards. This is a new perspective on measuring oral health and the OHRQoL improved with the treatment in participants with good perceived oral health. Second, these participants may not have recalibrated, but the DIT had a little or no influence in their appreciation of their oral health. Nonetheless, this interpretation should be viewed cautiously. Statistical analysis such as CRT can identify predictors but not meanings of oral health for the participants.

This study used the OHIP-Edent MID as threshold to identify clusters of participants potentially recalibrating. In this context, it should be acknowledged that MID is a conservative measure that may vary across populations and treatments [41] and may have left some recalibration undetected.

CRT also allows the investigation of reprioritization by exploring the importance of each variable to the model. At baseline and follow-up, physical disability was the most important variable, but the importance of physical pain decreased. The social disability and psychological discomfort aspects of QoL increased in importance over time as participants became more aware of the impact of these areas on their everyday life. Nonetheless, the increased importance between the baseline and follow-up assessments of one variable necessarily implies a decline on the other. Thus, the model must be interpreted carefully.

This is the first time that CRT has been used to assess RS in people with dental implants. One of its advantages is the graphical data representation. Clusters of participants with certain characteristics can be easily identified and this is important in analyses of large data sets. Nonetheless, trees are subject to large variance and slight changes in data might result in different trees. Based on these data, CRT is recommended as an effective approach to assess RS. This method does not require retrospective assessments; thus, it is not susceptible to recall bias, nor does it increase the burden on participants.

Compared to other statistical approaches to assess RS, CRT has potential. Structural equation modelling (SEM) has been extensively used to assess RS [42, 43]. Although SEM and CRT share some advantages (e.g. operationalizing all three aspects of RS, allowing the use of multiple data points and thus facilitating a direct interpretation of RS), CRT has several benefits. For example, CRT is able to detect RS even when its prevalence is low and allows RS identification at the individual level. On the contrary, SEM can only detect RS if it occurs in a minority of the outcomes, in a substantial portion of the sample and targets RS at the group level [7].

The results of the then-test, the self-anchored and the CRT approaches are compatible. Overall, the three approaches detected RS and showed similar directions. The then-test validated the self-anchored scale because both showed similar recalibration patterns, i.e. overall, participants changed their internal standards downwards. As CRT is not susceptible to recall bias, findings of this study suggest that both CRT and the then-test measure recalibration. Likewise, areas susceptible to recalibration are comparable between the then-test and the CRT. By definition, the observed change (follow-up minus baseline scores) is the same for both approaches. Functional limitation is apparently the most susceptible to recalibration as shown by the then-test and the CRT.

However, the comparison of approaches to assess RS is complex. The different methods assess different aspects of RS. Moreover, the different types of RS are likely to occur together and may cancel each other out [44]. The then-test used in this study detected recalibration, but is susceptible to recall bias. CRT detected recalibration and reprioritization, but is susceptible to large variance. Therefore, it is important to explore the convergent validity of complementary methods using several at once.

In terms of feasibility, the then-test was easily implemented and clearly understood by participants, but the self-anchored scale required an additional cognitive effort, which apparently caused a considerable data loss. This disadvantage severely limits its feasibility. Moreover, the self-anchored scale may be assessing another concept than RS. The CRT does not increase the burden on participants and is not subject to recall bias, as it does not require retrospective assessment. Therefore, the results of this study suggest that the then-test and CRT have good convergent validity, showing similar patterns of RS. This similarity might also indicate that the then-test was not subject to recall bias, as both methods use a statistically different operationalization of RS leading to the same result. Thus, when used together, both approaches complement each other to assess RS.

This study has limitations. It is restricted to implant treatment and the sample was unequally distributed regarding DIT modalities. Thus, replicating this study in a population receiving other treatment is required. With the absence of a gold standard for the duration of studies investigating RS, the follow-up period for these participants varied from 3 to 6 months. Thus, these findings need to be tested in studies with shorter and longer follow-up. Moreover, future research using larger samples should explore the role of other sociodemographic characteristics as predictor variables of RS.

This study shows that simple comparisons of OHRQoL scores at baseline and at follow-up (unadjusted change) might overestimate the benefits of dental impact treatment and that RS should be accounted for in such evaluations. These findings may also be useful in clinical care. Despite the benefits of using patient-reported outcomes and their wide recognition for providing important information, their use in healthcare and particularly in dentistry is not common. One of the major advantages of using assessments of quality of life is the evaluation of the benefits of the treatment from the individual perspective. However, there is some difficulty when interpreting scores changes. Frequently, when assessments of OHQoL are included, they do not reflect improvements in oral health that may have occurred if RS had been considered. Thus, health practitioners should incorporate quality of life measures routinely and RS is crucial. Providing patients with adequate information before treatment may manage their expectations and so maximize the treatment gains.

## Electronic supplementary material

Below is the link to the electronic supplementary material.

Supplementary material 1 (DOCX 23 kb)
