# Supplementary material for: How peoples’ ratings of dental implant treatment change over time?
Source: Qual Life Res. 2020 Jan 6;29(5):1323–34. doi: 10.1007/s11136-019-02408-1 (PMC7190585; doi:10.1007/s11136-019-02408-1)
Supplement: Supplementary file 1 — Supplementary material 1 (DOCX 23 kb) [file 11136_2019_2408_MOESM1_ESM.docx]

**Appendix 1**

Table 1. Missing data. Demographic characteristics

|  | Baseline  (N=127) | | Follow-up  (N=100) | | Missing  (N=27) | |
| --- | --- | --- | --- | --- | --- | --- |
|  | N | % | N | % | N | % |
| **Gender** |  |  |  |  |  |  |
| M | 54 | 42.5 | 38 | 38 | 16 | 29.6 |
| F | 73 | 57.5 | 62 | 62 | 11 | 15.1 |
| **Age** |  |  |  |  |  |  |
| 16-25 | 41 | 32.3 | 28 | 28 | 13 | 31.7 |
| 26-59 | 69 | 54.3 | 56 | 56 | 13 | 18.8 |
| >60 | 17 | 13.3 | 16 | 16 | 1 | 5.9 |
| **Type of treatment** |  |  |  |  |  |  |
| SDI | 99 | 78.0 | 74 | 74 | 25 | 25.3 |
| ISFPD | 13 | 10.2 | 12 | 12 | 1 | 7.7 |
| OD | 15 | 11.8 | 14 | 14 | 1 | 6.7 |

Table 2. OHIP-Edent baseline and follow-up missing data

|  | **OHIP-Edent Baseline scores** | | | | | | | |  |
| --- | --- | --- | --- | --- | --- | --- | --- | --- | --- |
|  |  | Functional | Physical | Psychological Discomfort | Physical Disability | Psychological Disability | Social Disability | Handicap | Total |
| **OHIP-Edent Total score Follow-up** | t | 2.0 | 2.3 | 2.0 | 2.3 | 1.1 | 1.7 | 1.8 | 2.5 |
|  | df | 48.6 | 48.5 | 43.4 | 42.8 | 38.8 | 52.2 | 50.0 | 48.4 |
|  | p(2-tail) | 0.05* | 0.02* | 0.05* | 0.02* | 0.28 | 0.09 | 0.07 | 0.01* |
|  | Present | 100 | 100 | 100 | 100 | 100 | 100 | 100 | 100 |
|  | Missing | 27 | 26 | 27 | 25 | 25 | 24 | 25 | 24 |
|  | Mean (Present) | 6.7 | 6.7 | 5.4 | 5.6 | 4.7 | 3.8 | 3.3 | 36.4 |
|  | Mean (Missing) | 5.4 | 4.7 | 4.5 | 3.9 | 4.1 | 2.7 | 2.4 | 27.9 |

* Statically significant at p<0.05
